# Exploring the Role of Green Finance and Energy Development towards High-Quality Economic Development: Application of Spatial Durbin Model and Intermediary Effect Model

**DOI:** 10.3390/ijerph19148875

**Published:** 2022-07-21

**Authors:** Rong Wang, Fayuan Wang

**Affiliations:** 1 Business School, Nanjing Xiaozhuang University, Nanjing 211171, China; rongwang@njxzc.edu.cn; 2 School of Economics and Management, Yangtze University, Jinzhou 434023, China

**Keywords:** green finance, energy development, high-quality economic development

## Abstract

Finance is the blood of the economy, and energy is the foundation and source of power for economic and social development. It is crucial to the prosperity and development of the country, the improvement of people’s lives and the long-term stability of society. It is a booster for the implementation of the concept of green development and the realization of high-quality economic development (HQED). Based on the panel data of 11 provinces and cities in the Yangtze River Economic Belt from 2007 to 2019, this paper selects green investment and carbon emission intensity as green financial values and calculates energy development indicators from the three dimensions of energy supply, energy consumption and energy efficiency. The three dimensions of development capability, high-quality development structure and high-quality development benefit are used to construct an indicator system for high-quality economic development, and the spatial Durbin model is selected to study the spatial effects of green finance and energy development on high-quality economic development. At the same time, the mediation effect model is used to test whether there is a mediation effect in the development of green finance on high-quality economic development. The results show that: green finance has a significant positive impact on high-quality economic development, and the spatial spillover effect is not significant; energy development has a significant positive impact on high-quality economic development, and the spatial spillover effect is significantly negative; the interaction term between green finance and energy development has a significant negative impact on high-quality economic development, and the spatial spillover effect is not significant and green finance plays a partial intermediary role in the process of energy development promoting high-quality economic development. Existing research considers less of the impact of green finance on high-quality development. On the one hand, the research in this paper can theoretically supplement and improve existing research and expand the research field; on the other hand, it can provide a policy basis for the realization of high-quality development in the region, which has important practical significance for the realization of sustainable development goals in the region.

## 1. Introduction

With industrialization in countries around the world, environmental pollution becomes a common danger threatening all mankind. It has become the common goal of all mankind to transform the types and methods of energy utilization, reduce environmental pollution such as carbon emissions and optimize the allocation of industrial structures [1]. It can be said that energy development is facing severe challenges and rare opportunities for transformation and upgrading [2,3,4]. China is a big manufacturing country and needs to be transformed into a strong manufacturing country, and it needs to move towards the high end of the manufacturing industry in the world. Energy is the food and blood of manufacturing industry [5,6,7]. The process of energy development is the process of the transformation of energy structure utilization. This is not only related to the transformation and upgrading of the real economy based on manufacturing, but it also involves the important strategy of “carbon-neutrality” and “carbon peak” [8]. Therefore, starting from the overall situation of environmental protection, the energy structure needs to be transformed from fossil energy to clean energy. With the transformation of energy structure, China is vigorously developing green technology innovation, advocating green finance and fully supporting the transformation of manufacturing from quantity to quality [9,10]. During the “14th Five-Year Plan” period, under the new situation that China’s external demand is under increasing pressure and regional development is facing huge challenges, how to promote coordinated regional development has become the key to achieving high-quality economic development. High-quality development is a development that is oriented and aimed at breaking the constraints of resources and environment and meeting people’s needs for a better ecological environment. Obviously, to promote the high-quality development of the regional economy, it is necessary to improve the environmental quality. The Yangtze River Economic Belt is the main force leading the HQED [11,12]. As the city group with the highest degree of regional integration in China, the Yangtze River Delta has a continuously expanding population, and the contradiction between ecological environment pollution and economic development has become increasingly prominent. Thus, are there differences in the role of high-quality development in the Yangtze River Delta in terms of its own characteristics and spatial laws? Furthermore, what is the mechanism by which energy development affects the high-quality economic development of the Yangtze River Delta? Clarifying the above problems will not only provide new policy perspectives for local governments to promote high-quality economic development in the Yangtze River Delta, but it will also provide valuable experience for reference in the design of environmental governance policies for other urban agglomerations.

At the same time, as a bridge and link, green finance provides important support for HQED. With industrial development and environmental protection, Salazar (1998) [13] believes that in areas highly dependent on resources, green finance is the link between ecological environmental protection and the connection between green industry and financial industry and it is a financial innovation based on the ecological environmental protection industry. Green finance focuses on the environmental pollution and ecological protection, etc., problem [14,15]. HQED has great demand for green investment, green financial tools and green ecological industries. Green finance accelerates regional economic structure adjustment through the interaction and benefit transmission mechanism at the public, enterprise and industry levels [16,17], thereby providing an important guarantee for accelerating high-quality economic development. Therefore, green industries can develop green economy even more powerfully, and social resources can be better allocated to create more high-quality economic growth points [18,19]. Then, how does the impact mechanism of green finance work? What kind of real effects there are and how to optimize green financial policies to promote high-quality economic development constitute important research topics. Therefore, it is worth further exploring green finance’s effect.

Based on the above background, the article uses the spatial Durbin model to verify the spatial spillover effect between variables and analyzes the green finance intermediary effect. The article first sorts out the existing related research and then introduces the selection of variables and the setting of the model. On this basis, it conducts an empirical analysis of spatial effects based on the data of the Yangtze River Economic Zone and based on the different regions. We compare the analysis results with existing research and carry out discussions and finally draw the conclusions of this research and put forward prospects for further research.

## 2. Literature Review

Green finance is not only a manifestation of industrial structure level but also a measure to improve the industrial structure upgrading. Energy development includes the transformation of energy utilization from fossil energy to clean energy, and its essence is still the changes brought about by industrial structure upgrading. Observe the existing research results, mainly in three aspects:

(1) Green finance impact on industry and economic development

Green finance is born with the requirements of industrial structure upgrading because of its selective investment direction which focuses on green industries. Qiao et al. (2021) [20] thought that green finance has an effective intermediary and adjustment role in national environmental regulations and policies and can promote enterprise innovation. Xie (2021) [21] found that green finance development positively regulates the improvement effect of environmental regulations on corporate technological innovation. Wang and Wang (2021) [22] research shows that green credit could effectively promote the green innovation of enterprises. Zhu et al. (2021) [23] researched that green finance impacts are affected by environmental policies and R&D investment, and there are industry and regional differences. Wang (2021) [24] believes that the green finance is relatively low and the green policy in the fiscal sector has no significant effect with green finance. The research of Zhou et al. (2021) [25] shows that green finance can improve economic structure, promotes economic innovation and development, promotes economic green development, inhibits stable economic development and has no significant impact on economic efficient development. Zhang (2021) [26] researched that green finance and carbon trading methods are the starting point for achieving “carbon-neutral” policies, which can allocate resources and leverage financial resources to tilt toward low-carbon green projects.

(2) Energy development impact on HQED

In the process of industrialization to post-industrialization, the energy structure is changing from fossil energy to clean energy. Luo (2019) [27] researched that the input of energy factors can provide sustainable support for economic development. Tang et al. (2020) [28] thought that energy consumption models development could reduce enterprise production costs and price levels, increase output and consumption and increase employment and investment. Wang and Wang (2019) [29] based on data from across China believe that energy investment and economic growth have a close relationship. Dong (2020) [30] believes that in the high-speed and non-high-speed periods, industrial economic growth and energy have a two-way impact. However, the research conclusion of Du et al. (2020) [31] showed that the energy consumption impact on the economy is slowly decreasing. From the perspective of the effects of changes in the energy structure, Dogan and Seker (2016) [32] and Zoundi (2017) [33] showed that the active development of clean energy can effectively reduce carbon emissions. However, Kahia et al. (2016) [34] found that clean energy has no significant effect on carbon dioxide emission reduction. Liu and Yang (2020) [35] found that technological progress and energy consumption present dynamic non-linear characteristics with economic development. Xu et al. (2020) [36] researched that there is a significant and stable dual-threshold effect between energy structure, ecological environment and economic development. Xu et al. (2019) [37] showed that clean energy effects are extremely heterogeneous, the impact on carbon dioxide emissions is non-linear and energy development impact on economic growth is also heterogeneous, with a gentle “W-shaped” non-linear impact on the eastern and central regions and an “inverted U-shaped” model for the non-linear impact on economic growth in the western region. Fu et al. (2021) [38] suggested that the synergy between energy security and HQED needs to be handled in a coordinated manner and coordinated to promote energy security, economic and social development and regional coordinated development.

(3) Research on high-quality economic development

Although foreign cities do not have the term “high-quality”, they have also done a lot of work in improving the quality of economic development. In terms of research on improving the overall economic development quality of urban agglomerations, the Atlantic coastal urban agglomerations in the northeastern United States have eased the pressure of population agglomeration in key cities by relying on a multi-level population development pattern and have used a sound industrial hierarchy to achieve dislocation and different quality among cities. Therefore, an urban agglomeration that can make full use of their respective characteristics and develop in coordination with each other is formed, and through the interaction between “government–non-government-market”, the rational allocation of resources and the coordinated development of regions are arranged more rationally. Japan’s Pacific coast urban agglomerations make full use of the interaction between the market and the government. The guiding role is to automatically and effectively allocate the population and resources in the region, so as to achieve mutual coordination between the entire urban agglomeration; the Pacific coast urban agglomeration makes full use of the existing advantages of each city to reasonably divide the labor in different cities and maximize the utility of the elements, at the same time solving the problem of urban development. Thomas (2000) [39] began to study the quality of growth associated with high-quality economic development in his book “Quality of Growth”; Barro’s (2002) [40] study emphasized the quality aspects of economic development, including health, fertility, income distribution, political institutions, crime, religion, etc.; Mlachila (2017) [41] pointed out through empirical investigation that the main factors of growth quality are external factors such as political stability, public expenditure on poverty alleviation, macroeconomic stability, financial development, institutional quality and foreign direct investment.

In the past two years, domestic scholars have made a lot of discussions on the development quality of cities and regions, using data processing methods and different index evaluation systems to conduct related research. Liu and Han (2021) [42] found that there existed positive effects between innovation efficiency, two-way openness and high-quality development. Lin et al. (2021) [43] has shown that information and communication technology has an inverted U-shaped influence on economic development and technology diffusion and talent allocation play a non-linear regulatory role in it. Hu et al. (2021) [44] researched that increasing the proportion of investment in environmental governance, actively absorbing public participation and strengthening support for environmentally friendly companies could improve high-quality economic development and have different effects in different regions. Wei et al. (2021) [45] showed that urban low-carbon governance construction affects green economic growth through measures such as environmental policy strength, urban carbon emissions, energy efficiency, industrial structure and green technology innovation.

Looking at the existing research literature, different scholars have studied the effect and mechanism of green finance and energy development from multiple perspectives. It is the entry point and one of the innovation points of the research in this paper. In addition, the existing research rarely studies the mediating effect of variables. This paper explores the green finance mediating effect. Achieving HQED is of great practical significance. Thirdly, there are many existing studies on the national level and few studies from an economic belt. This paper takes the Yangtze River Economic Belt as an example to explore the issue of HQED. It will be a useful supplement to existing research.

## 3. Method

Due to the objective economic or social connection between regions, the economic, social and environmental indicators between regions have mutual influences in space. In this context, the sample data do not necessarily meet the assumptions of an independent and identically distributed normal distribution, so it is necessary to consider the use of spatial econometric models to analyze the impact of various factors on high-quality development.

### 3.1. Model Setting

Spatial substantive correlation and spatial perturbation correlation are two manifestations of spatial correlation. When there is the phenomenon of element flow and diffusion, the expression of variables in one area may affect another area, forming a spillover effect, which is manifested as a substantial spatial correlation. However, if the impact on other regions is not related to the role of its key variables but is caused by random interference terms, then the spatial correlation is spatial perturbation correlation. Spatial panel models are divided into the spatial lag model (SLM), spatial error model (SEM) and spatial Durbin model (SDM). The spatial lag model can measure the influence of the explained variable in a certain area on the explained variable in the adjacent area. The spatial error model is used to explore the influence of the adjacent regions of a certain region on the explained variables of the region due to the impact of the spatial error term. When the spatial lag term of the explanatory variables has an impact on the explained variables, the establishment of a spatial Durbin model should be considered [46,47]. The spatial econometric model can well explain the spatial dependence between different variables in the observation unit relation. The spatial Durbin model includes the spatial correlation between explained variables and explanatory variables, that is, the explained variables in a certain area can be affected by the explained variables and explanatory variables in the adjacent areas [48]. We construct the spatial model as follows:

$${HQED}_{it}{=\alpha }_{0}{+\alpha }_{1}{GF}_{\mathrm{it}}{+\alpha }_{2}{ED}_{it}{+\alpha }_{3}{GF\_ED}_{it}{+\alpha }_{4}{Col}_{it}{+\alpha }_{5}W_{ij}*{GF}_{it}{+\alpha }_{6}W_{ij}*{ED}_{it}$$

(1) $${+\alpha }_{7}W_{ij}*{GF\_ED}_{it}{+\alpha }_{8}W_{ij}*{Col}_{it}{+\mu }_{i}{+v}_{t}{+\varepsilon }_{it}$$

In Formula (1), ${HQED}_{it}$, ${GF}_{it}$, ${ED}_{it}$ and ${GF\_ED}_{it}$ respectively represent i province and city of HQED, green finance development level, energy development and green finance integration and energy development in year t, and ${Col}_{it}$ is the status of the control variables of *i* province and city in year *t*. $\alpha_{i}$ is the coefficient needed to estimate,$\;\mu_{i}$ represents spatial fixed effect, $v_{t}$ represents time-point fixed effect,$\;\varepsilon_{it}\;$represents error term, and *W_ij_* represents spatial weight matrix. The paper selects the spatial weight matrix of economic distance with per capita GDP into the model for test analysis:

(2) $$W_{ij}=\{\begin{array}{cc} 1/|\bar{y_{i}}-\bar{y_{j}}| & \;\&i\neq j\\ 0 & \;\&i=j \end{array}$$

In the above formula, $\bar{y_{i}}$ and $\bar{y_{j}}$ are the per capita GDP of area *i* and area *j*.

### 3.2. Spatial Autocorrelation Test

Before using a spatial econometric model to analyze how green finance and energy development affect high-quality development, it is first necessary to examine whether there is a spatial autocorrelation in the high-quality development of the dependent variable. If it is found through inspection that high-quality development objectively has autocorrelation characteristics, it is necessary to consider spatial factors when using the model for regression analysis; otherwise, the estimation results are prone to inconsistency. Therefore, this paper uses Moran’s I index method to test the global spatial correlation of high-quality development and measures whether there is a spatial correlation in the geographical distribution of high-quality development. The value range of Moran’s index is (−1, 1), and if Moran’s index is greater than 0, this indicates that each region presents a positive spatial correlation. If Moran’s index is less than 0, this indicates that each region has a negative spatial correlation. If the Moran index is 0, this indicates that there is no spatial correlation. The calculation method of Moran’s *I* is as follows:

(3) $$I=\frac{\sum_{i=1}^{n}\sum_{j=1}^{n}w_{ij}\left(Y_{i}-\bar{Y}\right)\left(Y_{j}-\bar{Y}\right)}{S^{2}\sum_{i=1}^{n}\sum_{j=1}^{n}w_{ij}}$$

In the formula, *X_i_* is the observed value, $S^{2}=\frac{1}{n}\overset{n}{\underset{i=1}{\sum }}{\left(X_{i}-\bar{X}\right)}^{2},\bar{Y}=\frac{1}{n}\overset{n}{\underset{i=1}{\sum }}X_{i}$ and w_ij_ is the element value of the space weight matrix. Moran’s *I* ∈ [−1, 1]. If Moran’s *I* < 0, this means negative spatial correlation; if Moran’s *I* = 0, this means no correlation; if Moran’s *I* > 0, this means a positive spatial correlation.

### 3.3. Model Selection Related Tests

The LM test is a common test method used to judge whether to choose SLM or SEM. This test is based on the OLS test to test the significance of the LM-error and LM-lag statistics. If neither LM-error nor LM-lag rejects the null hypothesis, it can be considered no spatial effect and the OLS model continues to be selected; if LM-lag rejects the null hypothesis but LM-error does not reject the null hypothesis, choose SLM; otherwise, choose SEM; if both LM-lag and LM-error reject the null hypothesis, then perform the robust LM-test (robust pull Grange multiplier test), if one of robust LM-error and robust LM-lag does not reject the null hypothesis, the results of the LM test are used, and if both of them reject the null hypothesis, then it is considered that SDM can be used for the construction model [49].

### 3.4. Mediating Effect Model

**Figure 1:**
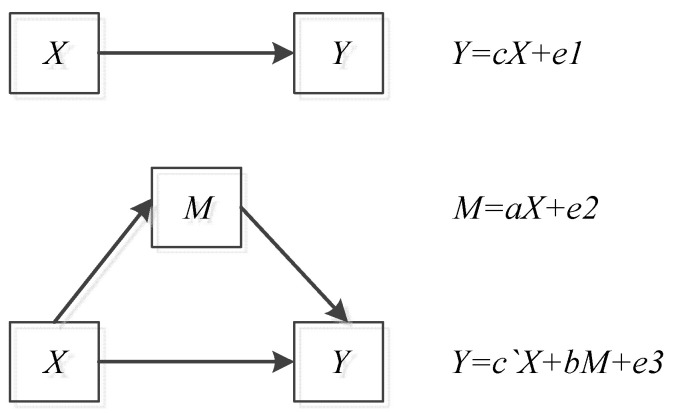
Relationship of *X*, *Y* and *M*.

The mediating effect model is to study whether there is a mediating effect between variables, and it is often used by scholars to study the relationship between the three variables. Specifically, if *X* affects *Y* by *M*, then *M* is the mediating variable. The relationship of *X*, *Y* and *M* is as follows in Figure 1.

In Figure 1, *ei* is a random error. If a, b and c are significant, it indicates that there is a mediating effect and the mediating effect accounts for ab/c in the total effect. Moreover, if c` is also significant, it means a partial mediation effect; if c′ is not significant, it means a complete mediation effect, the following spatial Doberman model with fixed time effects is constructed:

(4) $${HQED}_{it}=\tau_{0}+\tau_{1}{ED}_{it}+\;\tau_{2}{Col}_{it}+\tau_{3}W_{ij}*{ED}_{it}+\;\tau_{4}W_{ij}*{Col}_{it}{+v}_{t}{+\varepsilon }_{it}$$

(5) $${GF}_{it}=\;\varphi_{0}+\varphi_{1}{ED}_{it}+\varphi_{2}{Col}_{it}+\;\varphi_{3}W_{ij}*{ED}_{it}+\varphi_{4}W_{ij}*{Col}_{it}{+v}_{t}{+\varepsilon }_{it}$$

$${HQED}_{it}=\;\beta_{0}+\beta_{1}{GF}_{it}+\beta_{2}{ED}_{it}+\;\beta_{3}{Col}_{it}+\beta_{4}W_{ij}*{GF}_{it}+$$

(6) $$\beta_{5}W_{ij}*{ED}_{it}+\beta_{6}W_{ij}*{Col}_{it}{+v}_{t}{+\varepsilon }_{it}$$

In the above formula,$\;\tau_{i},{\;\varphi }_{i}$ and $\beta_{i}$ represent the coefficients needed to estimate, $v_{t}\;$represents the fixed effect and $\varepsilon_{it}\;$represents a random error. Obviously, in Formulas (4)–(6), the coefficients to be estimated $\varphi_{1}$, $\beta_{2}$, $\tau_{1}$ and $\beta_{1}$ respectively represent the impact of energy development on HQED in the development process, and green finance is used as a mediating variable in the mediation effect model of a, b, c and c′. Thus, Equations (4)–(6) constitute a complete mediation effect test model.

## 4. Results and Discussion

According to the selection of research methods in the previous article, this paper conducts an empirical analysis on the basis of variable selection and discusses the differences and connections between the existing results and previous scholars on this basis, in order to highlight the innovation of this paper.

### 4.1. Variable Selection

(1) Explained variable

High-quality economic development (HQED). The article constructs an economic high-quality index covering three first-level indicators and 13 s-level indicators. The development index system is shown in Table 1. Then, the negative indicators are converted into positive indicators by the reciprocal method, and the maximum value normalization method is used to carry out the dimensionless processing of all indicators. Finally, the United Nations Human Development Index is used for reference. The comprehensive index of quality development is calculated in detail, and the results are shown in Table 1. This method of empowerment may be somewhat arbitrary, but the purpose here is to emphasize that high-quality economic development involves many aspects.

Table 1 shows that in the comprehensive indicator system for high-quality economic development, the weights of capacity, structure and benefits are 0.4269, 0.2671 and 0.3060, respectively. It means that in the process of HQED, economic development capacity accounts the largest, followed by development benefits and finally development structure.

(2) Explanatory variables

Green finance (GF). Considering the completeness of the indicator setting and the availability of data, based on the connotation and service types of green finance, and referring to the setting of the green finance indicator system, the explanatory variables of green finance are divided into green credit, green securities, green investment, green insurance and carbon finance, which are indicators of five dimensions, and these five dimensions are synthesized into a comprehensive level indicator of green finance development by principal component analysis to consider their impact. The measurement of green credit generally has two types: forward and reverse. Here, the reverse index is used to measure, specifically, the proportion of the interest expenditure of the six high-energy-consuming industrial enterprises above the designated size in the total interest expenditure of the industrial enterprises above the designated size; the measurement of green securities, using the ratio of the A-share market value of environmental protection companies to the total market value of A-shares, is expressed; the measurement of green insurance is most appropriate for using the proportion of the environmental liability insurance scale. However, due to the late implementation of environmental liability insurance and insufficient data disclosure, the measurement of green insurance is related to more relevant agricultural insurance replacing environmental liability insurance) [50]; the measurement of carbon finance is expressed by carbon dioxide emissions/GDP; as for the measurement of carbon dioxide emissions, it is measured by the consumption of three major energy sources (coal, oil and natural gas). The specific calculation formula is: $co_{2}=w_{1}\alpha_{1}coal+w_{2}\alpha_{2}petroleum+w_{3}\alpha_{3}nututal-gas$, where $w_{1}$,$\;w_{2}$ and $w_{3}$ are coal, oil and the carbon emission coefficient of natural gas, which, expressed by the average value of the carbon emission coefficient of IPCC and the Energy Research Institute of the National Development and Reform Commission, are 0.7520, 0.5845 and 0.4465. $\alpha_{1}$, $\alpha_{2}$ and $\alpha_{3}$ are the standard coal conversion factors of 0.7143, 1.4286 and 1.3330. The data on green credit, green investment, green insurance and carbon finance come from the EPS database, and the green securities data come from the WIND database.

Energy development (ED). Regarding energy development measurement indicators, referring to existing research, starting from energy supply, consumption and efficiency, we construct a comprehensive evaluation indicator system with six indicators in three dimensions [51,52,53] and use the entropy method to evaluate each indicator and objective empowerment, with a view to comprehensively measure the energy development. The detailed index system is as follows in Table 2.

(3) Control variables

Technology market environment (TME). A mature technology market can provide a beneficial environment for the transformation and application of scientific and technological innovation achievements. This paper uses the ratio of technology market turnover to GDP to measure it.

Financial development (FD). Foreign scholars have made a clear description of the intrinsic essence of financial development, that is, a sound financial system can continuously improve the composition of social capital, reasonably allocate social resource elements and organize social savings and investment in an orderly manner [54]. In this process, the structure of the financial market has been continuously optimized and the scale and flow of financial market transactions have gradually increased. At present, the selection of financial development indicators is not uniform. This paper measures it from three dimensions of financial development: scale, efficiency and structure. When measuring the scale of financial development, McKinnon proposed to divide the broad money supply M2 by the nominal GDP to reflect the degree of economic monetization. The larger the ratio between the two, the larger the scale of financial development. From the perspective of stock and flow, GoldSmith proposed to divide the total financial assets by the nominal GDP to reflect the financial development scale. The larger the ratio between the two, the higher the financial correlation rate. Considering that China lacks specific statistical data for M2 and financial assets in various regions, the article selects the balance of deposits and loans of financial institutions in each region to represent the level of financial assets in each region and substitutes it into the calculation formula.

Education level (EL). Higher education includes factors such as scale, specialty and personnel. Therefore, based on educational theory and other relevant knowledge, this article reflects the development level of higher education from the scale of education [55]. The scale of higher education is represented by the proportion of general higher education funding to GDP.

Human capital (HC). Skilled human capital represents the professional knowledge and skills that an individual possesses to complete a specific job, and it is the embodiment of the technical level of workers in a country or region. Skilled human capital can find more effective and lower-cost ways to meet and expand market needs [56,57]. In the process of accumulation, the improvement of its own work efficiency will also lead to the production efficiency improvement of other production factors. Technical level and production capacity improvement have an important influence and are the backbone of independent innovation [58]. The main social actors corresponding to the carrier of skilled human capital are workers who are proficient in professional and technical knowledge. What they have is not only technology but the skills formed on this basis. Therefore, combined with the definition of skilled human capital and the availability of data, this paper takes the enrollment rate of colleges and universities as a proxy variable of skilled human capital.

Descriptive statistics are performed as follows in Table 3.

The data in Table 3 show that the level of high-quality economic development is relatively low, with an average value of 0.3675, and the maximum value is five times the minimum value, indicating that regional differences are large and further discussion is necessary. The standard deviation is 0.1714, and the maximum and minimum values are 0.8436 and 0.1634, respectively. It can be seen that the level of high-quality economic development in the 11 provinces and cities in the Yangtze River Economic Belt is not high, and there is still a large gap between provinces and cities. The average values of green finance and energy development are 0.4751 and 0.3553, respectively, which also shows that the development level of green finance and energy in the Yangtze River Economic Belt is relatively low. Considering the situation of each control variable at the same time, it can be found that there are still large gaps in the development of provinces and cities in the Yangtze River Economic Belt as a whole and there are problems of unbalanced and insufficient development.

### 4.2. Test Process

(1) Results of spatial autocorrelation test

The paper chooses the Stata 15.0 software to measure Moran’s I index, with the results as follows in Table 4.

In Table 4, from 2007 to 2019, Moran’s I was significantly positive at the 1% test level, which indicated that the high-quality economic development of the 11 provinces and cities in the Yangtze River Economic Belt had a significant positive spatial correlation within the study interval. Therefore, it is an appropriate choice to choose a spatial econometric model to empirically analyze the spatial effect of high-quality economic development.

(2) Model selection related tests

The *p* values of LM-error and LM-lag are both less than 0.01, that is, the null hypothesis is rejected and a robust LM test is required. The robust LM test results show that the *p* values of robust LM-error and robust LM-lag are both less than 0.1, that is, the null hypothesis is rejected. From this, it can be preliminarily considered that the spatial Doberman model can be selected when modeling. In order to ensure that the results are more rigorous, the likelihood ratio test (LR test) and Wald test are also required to determine the type of model [59,60]. The test results are in Table 5.

### 4.3. Test Results

(1) Spatial Durbin model test results

The paper uses the Stata 15.0 metrology software. The results are shown in Table 6.

It can be seen that all variables have impacts on the high-quality development of the economy, and the test results are analyzed in detail below.

(2) Test results of intermediary effect.

The Stata measurement software is used to estimate Formulas (4)–(6) to test the intermediary role of green finance in the process of energy development affecting high-quality economic development (Table 7).

As shown in Table 7, model (3) shows the impact of energy development on high-quality economic development, model (4) shows the impact of energy development on green finance and model (5) shows the impact of green finance on high-quality economic development. In the influence of quality development, the result of the mediating effect is exerted and the proportion of the mediation effect in the total effect is ab/c.

### 4.4. Discussions

(1) A positive effect exists between green finance and HQED, but no spillover effect exists. The green finance coefficient is 0.1845 and passes the 10% test. It means that green finance could significantly promote HQED. In the process of lending to support the development of industries, the corresponding industries and enterprises are selected accurately. Therefore, it is conducive to supporting industrial structure upgrading. The W*GF index coefficient is −0.2197 but not significant, meaning that the green finance in one region is not closely related to neighboring region. Comparing existing studies, we find no literature on the spatial effects of these research areas. It is one of the innovations of this research.
(2) There also exists a positive effect between energy development and HQED, with negative spatial spillover effect. The direct impact of energy development is 0.5787, which is significant through the 1% test. This means the energy development of this area can significantly promote the HQED of this area because energy development means a shift from fossil energy to clean energy in terms of energy utilization. The application of clean energy not only improves energy efficiency but also improves air and environmental quality, which is beneficial for HQED. These are the same results as Dogan & Seker (2016), Zoundi (2017) and Du et al. (2019). The coefficient of the W*ED indicator, which means significant energy development spatial effect, is −1.104. It means that the development of energy has a significant negative impact on neighboring regions. This is because the pace of energy reform is still inconsistent. The vigorous use of clean energy in this province and city may attract high-quality resources from surrounding provinces and cities. Furthermore, the more polluting fossil energy may also flow into surrounding provinces and cities. Furthermore, the state supports energy reform, and its support to provinces and cities is also inconsistent. In the case of limited national project support funds, strong support for the energy reform of the provinces and cities will increase the corresponding project funds and the project funds supported by neighboring provinces and cities may inevitably decrease. The combination of these factors results in a significant negative effect of energy development space.
(3) The interaction term of green finance and energy development has a significant negative impact, and spatial effect does not pass the test. The GF_ED coefficient is −0.6102 and passes the significance test. It shows that the interaction between green finance and energy development has a significant negative impact on high-quality economic development. It may be because green finance is mainly reflected in the environmental protection investment ratio, as well as energy consumption and efficiency. The combination of the two squeeze funds for industrial development is not conducive to enterprise innovation investment and may affect the application of new achievements in industrial development. Therefore, it is harmful to HQED. Furthermore, the W*GF_ED coefficient is 0.7938 but not significant. Due to the environmental protection investment and energy consumption of this province and city, it can hardly affect the surrounding provinces and cities. This result has not been found in existing studies, and it is also one of the innovations of this research.
(4) Green finance plays a part of the intermediary effect in the process of energy development promoting HQED. The test results of the intermediary effect model show that in model (3), the coefficient (c) of energy development is significant with 0.3421, proving that energy development can improve high-quality economic development. In model (4), the energy development coefficient (a) is 0.1349, passing the 10% significance level, indicating that energy development also has a positive impact on green finance. In model (5), the energy development coefficient (c) is 0.2886 and the green finance coefficient (b) is 0.2293, and both pass the test level, indicating that energy development and green finance have high-quality effects on the economy. The development has a significant positive impact, which is consistent with the spatial Durbin model test of the double fixed effects, which proves the robustness and reliability of the results. Based on the above results, it shows that green finance plays a part of the intermediary effect in the process of energy development promoting HQED. The intermediary effect accounts for 0.1349*0.2293/0.3421 = 9.04% of the total utility. This result has not been found in existing studies, and it is also one of the innovations of this research.

To sum up, it can be seen that breaking the high-yield-high-pollution dilemma and exploring a high-quality development path of high-yield-low-pollution is of typical significance for realizing the high-quality development of the Yangtze River Delta region and my country’s overall economy. Based on this, starting from the financial system, we study the relationship between green financial investment, energy development and high-quality economic development and then explore the path of high-quality economic development from the perspective of financial institutions and promote the transformation and upgrading of regional industries. It has important reference significance for the high-quality economic development of other regions.

## 5. Conclusions

According to the above empirical analysis, this paper summarizes and analyzes the research results and puts forward the shortcomings and prospects of the research, hoping to provide a theoretical basis for the research and provide a reference for the government’s policy formulation.

### 5.1. Research Conclusions

(1) A positive effect exists between green finance and HQED, but the spatial spillover effect does not pass the test, and the green finance impact coefficient is significant with 0.1845, that is to say, there exists a significant direct positive effect. Combined with existing research, the mechanism is that green finance improves industrial structure upgrading, promotes enterprise innovation and technological transformation and thus promotes high-quality economic development. Furthermore, it shows that the spatial spillover effect coefficient of green finance is −0.2197 but does not pass the test. Combined with the comprehensive analysis of existing research, it is mainly because the support of green finance is limited in the province and city, and the driving effect on the surrounding area has not yet formed.
(2) Energy development also has a positive effect on HQED, and the spatial spillover effect is significantly negative. The direct impact of energy development is 0.5787, which is significant when it passes the 1% test. Based on existing studies, it is believed that energy development has been gradually eliminated due to the gradual elimination of fossil energy and the increase in the use of clean energy, which has promoted HQED. It means that the W*ED index coefficient of the space effect of energy development is −1.104. According to the analysis of relevant economic theory, it may be that the energy development of this province and city can attract high-quality resources to the province and city through the siphon effect, which will have a negative spatial spillover effect on the surrounding regions.
(3) The interaction between green finance and energy development has a significant negative impact on HQED, and the spatial spillover effect is not significant. The GF_ED coefficient is −0.6102, and this shows that the interaction between green finance and energy development has a significant negative impact. According to relevant economic theories and economic development practice analysis, green finance effectively supports clean energy development. It may squeeze the funds required for industrial innovation and upgrading. In addition, energy development is more manifested as an increase in energy consumption. It will inevitably squeeze funds for industrial development. The superimposed consequences of the two will affect HQED.
(4) Green finance plays a part of the intermediary effect in the process of energy development promoting HQED. The intermediary effect model shows that energy development promotes HQED and relies on the intermediary role of green finance. The finance intermediary effect accounts for 9.04% of the entire effect of energy development in promoting high-quality economic development. It means that in the process of promoting HQED, we can pay more attention to the intermediary role of green finance. To achieve HQED, it is not enough to rely solely on energy development. It is necessary to use macroeconomic policies to regulate and control and to play the combined role of multiple influencing factors.

### 5.2. Recommendation

According to the above analysis and conclusions, this paper explores the HQED suggestions of the Yangtze River Economic Belt from the following aspects:

(1) Enhance the ability of green finance to support high-quality development

Bank depository financial institutions gradually strengthened green credit support for green environmental protection industries, which can promote the development of green finance. Financial institutions set up special green departments to promote the development of green credit business, issued green credit guidelines and credit policies for green industries, such as photovoltaics, energy conservation, environmental protection and new energy vehicles, and actively carried out green labeling and classification management. Take green credit as the starting point, at the same time accelerating the innovation of green financial products, expanding green financial channels, forming an organic unity with green credit as the starting point, green insurance and green bonds developing together and effectively improving the green finance in the Yangtze River Economic Belt to support HQED ability.

(2) Establish a multi-party interconnection and interaction mechanism to vigorously develop green consumption

We must leverage green development from consumer terminals, regulating the green transformation of production models and even industrial systems. First of all, we must increase the high-quality development of consumer entities from two dimensions, continuously improve the HQED mechanism for internal and external linkages of consumer entities, explore key areas of green consumption and form a “government–enterprise–consumer” benign high-quality development linkage network. It is also possible to promote green consumption by building a sound legal system for green consumption and promoting a green label and procurement system. The second is to promote publicity green consumption, improve green consumption awareness, adjust the structural contradictions of energy consumption and increase the guidance of green culture construction. Therefore, the development of green consumption is the interconnection and interaction of multiple subjects including the government. On the basis of the protection of green consumption legislation, consumers are guided through green policies and green consumption is developed from the two dimensions of green consumption supply and demand on high-quality economic development.

(3) Improve energy consumption structure and increase renewable energy consumption

The specific operations are as follows: in the underdeveloped areas in the west, small hydropower is established to ensure the normal living needs of the residents; in the eastern area, resources are relatively lacking and it is recommended to speed up the development of nuclear power, and the coastal islands with relatively sufficient wind tend to increase investment in wind power. The combination of nuclear power and wind power ensures normal production demand and normal use in residents; in areas with relatively high sunshine in Ningxia and Tibet in the west, large-scale solar power generation will be generated and the conversion rate of solar hot water and other energy conversion methods will be improved in general areas; in Tibet, where geothermal energy is relatively developed, it is recommended to combine geothermal energy with farming to promote planting and breeding.

(4) Further enhance the level of energy innovation and development

The lack of scientific and technological innovation level has seriously hindered the development of my country’s energy. For enhancing energy innovation and development level, it needs to construct an innovation system and improve the combination mechanism of “government, industry, academia, research and application” in the energy field. It must achieve breakthroughs in key core technologies, such as coal-fired power generation technology, natural gas hydrate exploration and development technology, new energy technology and independent complete sets of major equipment technologies, and achieve technological innovation and institutional innovation in the fields of integrated energy, energy storage, smart energy and global internet energy, gradually narrowing the gap with developed countries and achieving catch-up and leapfrog.

### 5.3. Shortcomings and Prospects

This paper focuses on the impact of green finance on energy development. Although some meaningful conclusions have been obtained, it is limited by the influence of various subjective and objective factors; this paper still has the following problems:

(1) The research period is short. The incompleteness of statistical data and the lack of indicators in earlier years restricted the upper limit of the research period. The earliest research year of this paper is 2008. At the same time, due to the lag in the publication time of various statistical yearbooks, the lower limit of the research period is limited, and the most recent research period for this paper is only up to 2020.
(2) The data are not rich enough. First, the data indicators selected in this paper refer to previous research results, but indicators reflecting high-quality development should be multi-faceted. The author knows that the indicators selected in this article are not enough to fully describe the level of high-quality development. Second, when using a spatial econometric model to study the spatial role of factors influencing high-quality development, qualitative factors, such as government policies, cannot be considered.
(3) In the selection of the indicator system, based on the previous research, this paper uses more representative indicators, but it is not comprehensive enough, and the existing research on high-quality development indicators involves less. There is a certain degree of subjectivity. At the same time, in terms of the influencing factors of high-quality economic development, this paper only selects a few typical influencing factors, but it is not comprehensive enough. In the future, more influencing factors can be selected to expand research in this field.

## Figures and Tables

**Table 1:** Comprehensive evaluation index system.

Dimension Layer	Index Layer	Unit	Attributes	Weights
Ability (0.4269)	GDP growth rate	%	+	0.0499
	Social labor productivity	Ten thousand yuan/person	+	0.1022
	Per capita investment in fixed assets	yuan	+	0.0579
	Total retail sales of consumer goods per capita	yuan	+	0.0991
	Science and technology expenditure as a percentage of GDP	%	+	0.1178
Structure (0.2671)	The proportion of the secondary industry in GDP	%	−	0.0619
	The proportion of the tertiary industry in GDP	%	+	0.0670
	Population urbanization rate	%	+	0.0705
	Fiscal revenue as a percentage of GDP	%	+	0.0677
Benefit (0.3060)	GDP per capita	yuan	+	0.1138
	Per capita income ratio between urban and rural areas	/	−	0.0446
	Urban registered unemployment rate	%	-	0.0796
	Resident Engel coefficient	%	-	0.0680

**Table 2:** Comprehensive evaluation index system for energy development.

Dimension Layer	Index Layer	Unit	Attributes	Weights
Energy supply (0.2626)	Energy consumption per capita	Tons of standard coal	+	0.2626
Energy consumption (0.5941)	Coal consumption	Ten thousand tons	−	0.2587
	Electricity consumption	Billion kWh	+	0.2739
	Natural gas consumption	One hundred million cubic meters	+	0.0615
Energy efficiency (0.1433)	Energy consumption per unit of GDP	Tons of standard coal/ten thousand yuan	−	0.0744
	Electricity consumption per unit GDP	KWh/CYN	−	0.0689

**Table 3:** Variable descriptive statistics.

Variable	Observed Value	Mean	Standard Deviation	Minimum	Maximum
High-quality economic development (HQED)	143	0.3675	0.1714	0.1634	0.8436
Green finance (GF)	143	0.4751	0.1443	0.0751	0.7533
Energy development (ED)	143	0.3553	0.1165	0.1891	0.6732
Technology market environment (TME)	143	0.8505	0.8683	0.0229	3.9895
Financial development (FD)	143	6.1120	2.8769	1.7533	17.2035
Education level (EL)	143	1.7933	0.4363	0.6655	2.6189
Human capital (HC)	143	59.0394	5.3398	44.0446	72.2957

**Table 4:** Economic high-quality global Moran index.

Year	I	Year	I
2007	0.238 ***	2014	0.328 ***
2008	0.191 ***	2015	0.303 ***
2009	0.219 ***	2016	0.215 ***
2010	0.205 ***	2017	0.192 ***
2011	0.223 ***	2018	0.182 ***
2012	0.250 ***	2019	0.160 ***
2013	0.301 ***		

Note: *** means *p* < 0.01.

**Table 5:** Model selection related test results.

Test Type	Null Hypothesis	Statistics	result
LM test	SEM	13.422 ***	*SDM*
	Steady SEM	33.329 ***	
	SAR	11.291 ***	
	Steady SAR	33.198 **	
Hausman test	Random effect	163.18 ***	Fixed effect
Wald test	SDM can be simplified to SEM or SAR	46.89 ***	*SDM*
LR test	SDM can be simplified to SEM or SAR	38.10 ***	*SDM*
		38.79 ***	
	Spatial fixed effect is better than double fixed effect	43.95 ***	Double fixed effect
	Point fixed effect due to double fixed effect	170.09 ***	

Note: *** means *p* < 0.01, ** means *p* < 0.05.

**Table 6:** Estimation results of spatial Durbin model.

Variable	Coefficient	z Value	Variable	Coefficient	z Value
Green finance (GF)	0.1845 *	1.66	W*GF	−0.2197	−0.98
Energy development (ED)	0.5787 ***	3.28	W*ED	−1.1041 ***	−2.69
GF_ED	−0.6102 **	−2.12	W*GF_ED	0.7938	1.25
Technology market environment (TME)	0.0382 ***	4.40	W*TME	−0.0081	−0.49
Financial development (FD)	0.0131 ***	3.11	W*FD	−0.0141	−1.64
Education level (EL)	0.0666 ***	3.05	W*EL	0.1218 **	2.25
Human capital (HC)	−0.0029 **	−2.15	W*HC	−0.0100 ***	−3.02

Note: ***, ** and * mean *p* < 0.01, *p* < 0.05 and *p* < 0.1.

**Table 7:** Test results of the intermediary effect of green finance.

Variable	High-Quality Economic Development (HQED) Model (3)	Green Finance (GF) Model (4)	High-Quality Economic Development (HQED) Model (5)
Energy development (ED)	0.3421 ***	0.1349 *	0.2886 ***
	(6.22)	(1.69)	(4.53)
Green Finance (GF)			0.2293 ***
			(5.43)
W*ED	1.7086 ***	1.0360 ***	1.3782 ***
	(10.38)	(4.08)	(8.06)
W*GF			0.3217 ***
			(3.28)
Col	√	√	√

Note: *** and * mean *p* < 0.01 and *p* < 0. 1.

## Data Availability

The data used to support the findings of this study are available from the corresponding author upon request.
